# Spinal Anaesthesia for Complete Molar Pregnancy With Biochemical Thyrotoxicosis and Ventricular Ectopy

**DOI:** 10.7759/cureus.101614

**Published:** 2026-01-15

**Authors:** Ryan Glaser, Jeffrey R Bolon, Jared C Brazington, Kealan N Dale

**Affiliations:** 1 Anaesthesiology, University of the Witwatersrand, Johannesburg, ZAF; 2 Emergency Medicine, Port Macquarie Base Hospital, Port Macquarie, AUS

**Keywords:** complete molar pregnancy, gestational trophoblastic disease, neuraxial anaesthesia, obstetric anaesthesia, phenylephrine, premature ventricular complexes, spinal anaesthesia, thyrotoxicosis, ventricular ectopy, β-human chorionic gonadotropin

## Abstract

Gestational trophoblastic disease (GTD) presents unique anaesthetic challenges due to the combined risks of haemorrhage, cardiopulmonary compromise, and endocrine dysfunction. Complete hydatidiform mole is frequently associated with markedly elevated human chorionic gonadotropin (β-hCG) concentrations, which may result in biochemical or clinical thyrotoxicosis through thyroid-stimulating hormone receptor activation.

Anaesthetic technique selection during uterine evacuation remains contentious. General anaesthesia (GA) is often favoured because it allows airway control and rapid escalation in the event of massive haemorrhage, yet it may provoke significant sympathetic activation, which is undesirable in thyrotoxic states or in the presence of ventricular arrhythmias. Conversely, spinal or regional anaesthesia avoids volatile agent uterine relaxation and airway manipulation, potentially reducing bleeding and allowing earlier recognition of complications (e.g., thyroid storm or cardiopulmonary distress), but requires meticulous prevention and treatment of sympathectomy-related hypotension.

We describe the anaesthetic management of a 20-year-old primigravida at 16 weeks’ gestation with a complete hydatidiform mole complicated by severe β-hCG elevation, biochemical thyrotoxicosis, and frequent premature ventricular complexes (bigeminy). Despite biochemical thyroid dysfunction, the patient remained clinically euthyroid, with no tachycardia and no clinical features of heart failure. The patient was haemodynamically stable and had preserved ventricular function. Following structured risk assessment, spinal anaesthesia was selected. Hypotension secondary to sympathetic blockade was proactively managed using a phenylephrine infusion to maintain arterial pressure while avoiding tachycardia.

This case demonstrates that neuraxial anaesthesia can be a safe and physiologically appropriate alternative to GA in carefully selected patients with molar pregnancy, when endocrine status, cardiovascular reserve, haemorrhage risk, and institutional readiness for conversion to GA are rigorously assessed.

## Introduction

Gestational trophoblastic disease (GTD) encompasses a spectrum of disorders arising from abnormal proliferation of placental trophoblast, ranging from benign hydatidiform mole to malignant gestational trophoblastic neoplasia (GTN) [1,2]. Complete hydatidiform mole is characterised by diffuse villous oedema, absence of fetal tissue, and excessive trophoblastic hyperplasia, typically accompanied by markedly elevated serum β-hCG concentrations [1,2]. Although the widespread use of ultrasonography has facilitated earlier diagnosis and reduced maternal morbidity, complete mole continues to be associated with significant systemic complications relevant to anaesthetic practice [1,3].

The multisystem effects of GTD are driven primarily by excessive β-hCG (beta-human chorionic gonadotropin) production. Beyond its role as a tumour marker, β-hCG exhibits intrinsic thyrotropic activity owing to its structural similarity to thyroid-stimulating hormone (TSH), resulting in thyroid hormone overproduction when present in sufficiently high concentrations [4-6]. Consequently, biochemical thyrotoxicosis is common in complete mole, while overt hyperthyroidism and thyroid storm, although now rare, remain well documented and potentially fatal complications [5-7]. Importantly, even asymptomatic biochemical thyrotoxicosis may alter cardiovascular physiology and influence perioperative risk [5].

Standard management of complete hydatidiform mole involves prompt uterine evacuation, most commonly by suction curettage, particularly in patients wishing to preserve fertility [1,2]. Anaesthetic technique selection for this procedure remains debated, as current literature does not mandate a single approach, making case-based evidence and institutional experience particularly relevant. General anaesthesia (GA) has traditionally been favoured because it allows airway control, controlled ventilation, and rapid response to haemorrhage or conversion to hysterectomy [8-10]. However, GA is associated with sympathetic stimulation during induction, laryngoscopy, and intubation, which may exacerbate thyrotoxic physiology or precipitate arrhythmias [7,11].

Neuraxial anaesthesia, by contrast, attenuates the surgical stress response and avoids airway manipulation, potentially offering physiological advantages in selected patients [10,12]. Nevertheless, spinal anaesthesia carries an inherent risk of hypotension due to sympathetic blockade, which may provoke reflex tachycardia and increase myocardial oxygen demand in patients with heightened adrenergic sensitivity [7]. Current GTD guidelines, such as FIGO (International Federation of Gynecology and Obstetrics) recommendations, focus on standardised staging, FIGO WHO risk scoring, serial β-hCG surveillance, and risk-adapted therapy (single-agent for low-risk, multi-agent for high-risk), with selective surgery or radiotherapy and a goal of fertility preservation [1,2]. However, they offer limited direction on anaesthetic technique for uterine evacuation, and practice is therefore largely shaped by clinician judgement and institutional experience [1,2].

This report explores the rationale for spinal anaesthesia in a patient with complete hydatidiform mole complicated by biochemical thyrotoxicosis and ventricular ectopy, with particular emphasis on structured risk stratification, haemodynamic management, and vasopressor choice.

## Case presentation

Clinical course

**Figure 1:**
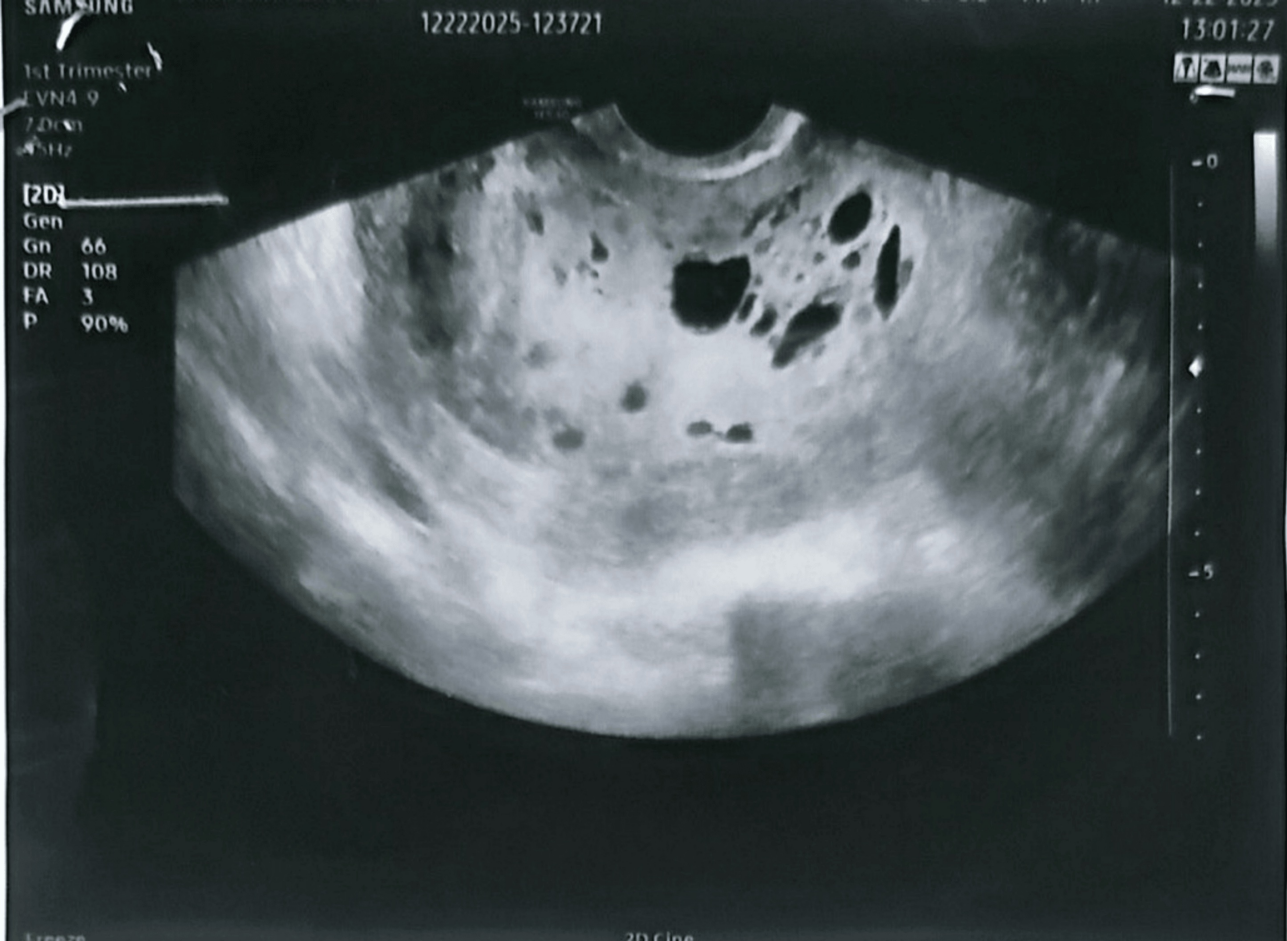
Transvaginal Ultrasound Demonstrating Diffuse Cystic Intrauterine Changes Without Identifiable Fetal Parts

**Figure 2:**
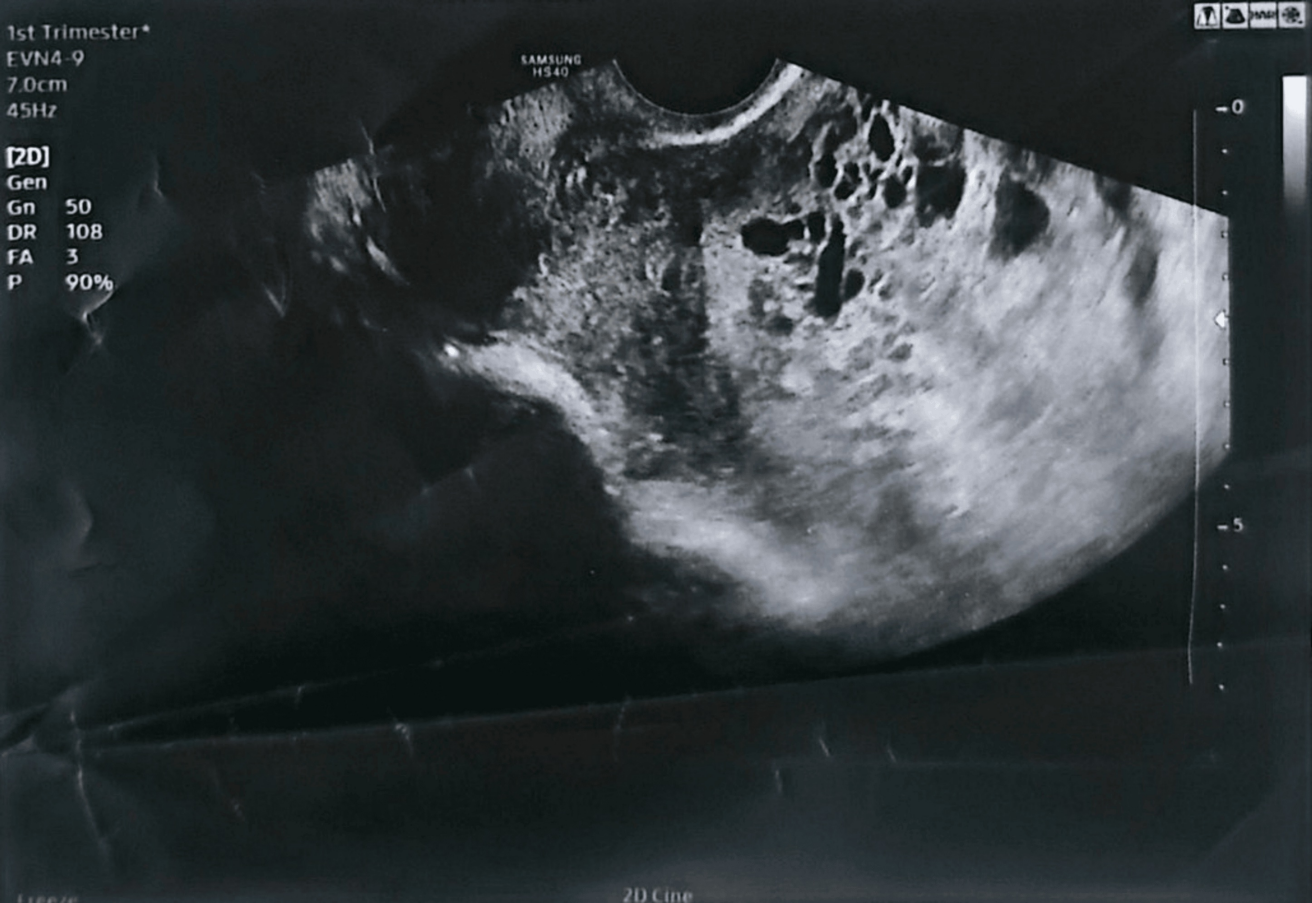
Transvaginal Ultrasound Showing the “Snowstorm” Appearance of a Complete Hydatidiform Mole

A 20-year-old woman, gravida 1 para 0, at 16 weeks’ gestation by dates, presented with a history of vaginal bleeding and abdominal distension. She reported progressive uterine enlargement and nausea but no vomiting, chest pain, dyspnoea, palpitations, weight loss, or heat intolerance. Ultrasound examination demonstrated a heterogeneous intrauterine mass with multiple anechoic cystic spaces and no identifiable fetal parts, consistent with complete hydatidiform mole, which is the classic "snowstorm" or "bunch of grapes" appearance described in GTD (Figures 1, 2).

Laboratory evaluation revealed a β-hCG concentration of 482,372 IU/L, in keeping with the marked hormone elevation typical of complete mole. Thyroid function tests showed suppressed TSH at 0.01 mIU/L with elevated free T4 at 28.0 pmol/L, fulfilling criteria for biochemical thyrotoxicosis, although the patient remained clinically euthyroid. Haemoglobin was 10.5 g/dL with a normal platelet count. Urea, creatinine, and serum electrolytes were within reference limits. Liver function tests were unremarkable.

A 12-lead electrocardiogram (ECG) demonstrated sinus rhythm with bigeminy premature ventricular complexes (PVCs). There was no ST-segment deviation or evidence of ischaemia. Transthoracic echocardiography showed a structurally normal heart with a left ventricular ejection fraction of 51% and no regional wall motion abnormalities.

**Figure 3:**
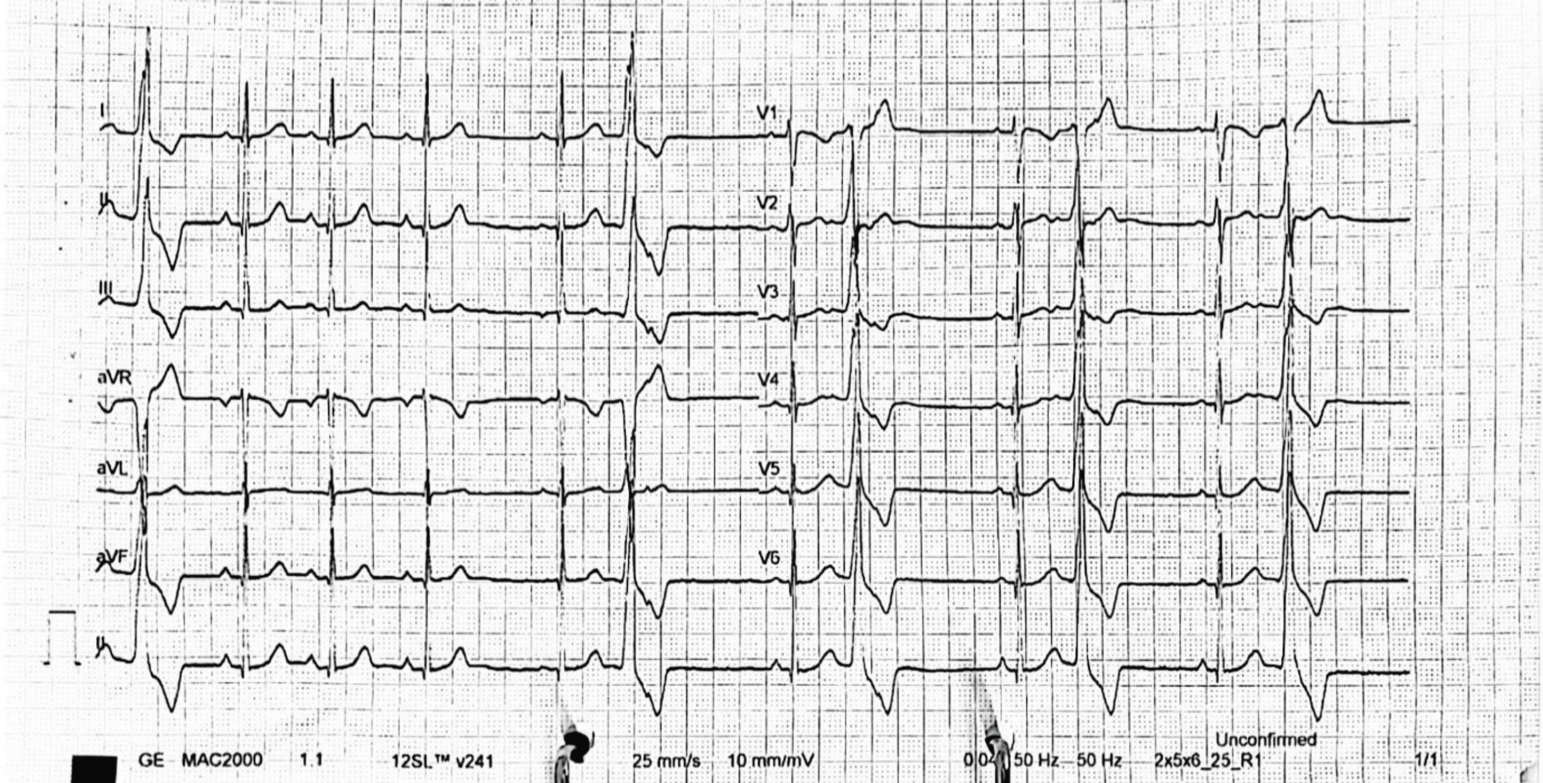
Electrocardiogram Demonstrating Sinus Rhythm With Ventricular Bigeminy Premature Ventricular Complexes Twelve-lead electrocardiogram demonstrating sinus rhythm with frequent premature ventricular complexes occurring in a bigeminal pattern, without ST-segment deviation or evidence of acute ischaemia.

These findings are consistent with ventricular ectopy in the absence of clinical features of structural heart disease, which is often well tolerated under anaesthesia when heart rate and haemodynamics are carefully controlled, particularly in young patients. However, vigilance is still warranted for progression to more significant arrhythmia (Figure 3).

On examination, she was afebrile with a heart rate of 82 beats/min, blood pressure 125/82 mmHg (mean arterial pressure 95 mmHg), respiratory rate 16 breaths/min, and oxygen saturation 99% on room air. There were no signs of heart failure or respiratory compromise. The thyroid gland was not palpably enlarged. There were no tremors, lid lag, or hyperreflexia. Using the Burch-Wartofsky score [7], her score was 0, which falls in the "unlikely to represent thyroid storm" category (<25), supporting the view that she had compensated biochemical rather than decompensated clinical thyrotoxicosis.

After multidisciplinary discussion between the anaesthesia and obstetrics teams, it was considered that GA would provide airway control and facilitate rapid response to haemorrhage but would expose the patient to laryngoscopy-induced sympathetic activation, which could aggravate ventricular ectopy and hyperdynamic thyroid physiology. Conversely, spinal anaesthesia would reduce adrenergic stimulation but carry a risk of hypotension due to sympathetic blockade. Given her clinical haemodynamic stability, preserved ventricular function, absence of heart failure, and an anticipated haemorrhage risk, with blood products available based on uterine size and preoperative assessment, spinal anaesthesia with invasive monitoring and immediate readiness to convert to GA was considered appropriate in this specific context. This should not be interpreted as a general recommendation for all GTD cases.

Anaesthetic management

The patient was fasted in accordance with standard obstetric anaesthetic guidelines. In the operating theatre, standard monitors were applied, including ECG with continuous arrhythmia detection, non-invasive blood pressure, and pulse oximetry. Before spinal anaesthesia, an awake radial arterial line was inserted under local anaesthesia for beat-to-beat blood pressure monitoring and blood sampling, given the potential for haemodynamic instability and arrhythmia risk. Two large-bore peripheral intravenous cannulae were sited. Two units of cross-matched packed red blood cells were reserved in theatre in anticipation of possible haemorrhage.

She received a preload of 700 mL of Ringer’s lactate, in line with common practice to mitigate spinal-induced hypotension while avoiding excessive fluid that could worsen occult cardiac dysfunction. Spinal anaesthesia was then performed at the L3/4 interspace using a 25-gauge pencil point needle with 1.4 mL of 0.5% hyperbaric bupivacaine combined with fentanyl 10 µg. A sensory block to T6 was achieved. Immediately after confirming block height, a phenylephrine infusion was commenced prophylactically at 12.5 µg/min and continued for 20 minutes (total dose approximately 250 µg). Phenylephrine was chosen due to its selective alpha-adrenergic effects, restoring systemic vascular resistance and arterial pressure without directly increasing heart rate, and is favoured over ephedrine where tachycardia would be undesirable [9,11]. Phenylephrine was started prophylactically because fluid therapy alone can have limited efficacy in preventing spinal hypotension, and effective management is best achieved with a proactive vasopressor strategy [9].

Blood pressure remained within 10% of baseline (target ≈125/82 mmHg, MAP 95 mmHg, based on ward vital-sign trends) throughout the procedure. The starting heart rate was 75 bpm, with an intra-operative range of 70-95 bpm, and there were no recorded changes in the frequency or pattern of ventricular ectopic beats during surgery. No significant hypotensive episodes occurred. Once haemodynamic stability was established, midazolam 1 mg was administered intravenously for mild sedation. Labetalol, magnesium sulphate, and amiodarone were prepared but not required.

The surgical duration was approximately 70 minutes. Estimated blood loss was 100-150 mL, and total crystalloid administration was 1600 mL of Ringer’s lactate. No uterotonic-refractory haemorrhage occurred, and uterine evacuation was completed without complication. There were no intra-operative episodes of bigeminy or other sustained arrhythmias. ECG monitoring remained stable without ischaemic changes. No signs or symptoms of thyroid storm developed. Serial arterial blood gases (ABGs) were obtained via the arterial line (Table 1).

**Table 1:** Perioperative Arterial Blood Gas Analysis Peri-operative arterial blood gas (ABG) trends and haemoglobin change. Serial ABG measurements taken pre-incision and at the end of surgery showed a stable, upper-normal pH with persistent hypocapnia (PaCO₂ 24→27 mmHg) and a mild metabolic base deficit (base excess −7.3→−5.1 mmol/L) that improved intra-operatively. Lactate remained normal (1.9→1.5 mmol/L), suggesting preserved tissue perfusion, and oxygenation was adequate (PaO₂ 94→118 mmHg). Haemoglobin decreased from 8.4 to 7.8 g/dL, consistent with mild intra-operative blood loss and/or dilution from fluid administration. Overall, the ABG trends support the suitability of neuraxial (spinal) anaesthesia in a stable patient with GTD.

Parameter	Pre-incision	End of surgery	Reference range	Interpretation
pH	7.45	7.44	7.35–7.45	Upper-normal pH
PaCO₂ (mmHg)	24	27	35–45	Respiratory alkalosis
PaO₂ (mmHg)	94	118	80–100 (room air)	Adequate oxygenation
HCO₃⁻ (mmol/L)	20.1	21.0	22–26	Reduced, suggesting metabolic compensation
Base excess (mmol/L)	−7.3	−5.1	−2 to +2	Metabolic acidosis component
Lactate (mmol/L)	1.9	1.5	<2.0	No tissue hypoperfusion
Haemoglobin (g/dL)	8.4	7.8	11.5–15.5	Progressive anaemia intra-operatively, likely dilutional

The mild respiratory alkalosis was consistent with pregnancy-related changes and light hyperventilation. Lactate decreased from 1.9 to 1.5 mmol/L, suggesting preserved tissue perfusion and absence of significant circulatory compromise. The modest drop in haemoglobin likely reflected haemodilution from crystalloid administration rather than frank blood loss, in keeping with the estimated 100-150 mL surgical loss. Serum sodium (132 mmol/L), potassium (4.0 mmol/L), and ionised calcium (1.25 mmol/L) remained unchanged and within acceptable ranges.

Postoperatively, the patient was transferred to an obstetric high-care unit for close monitoring of haemodynamics, thyroid status, and uterine bleeding. She remained stable, with no delayed arrhythmias or respiratory compromise. Plans were made for serial β-hCG measurements until normalisation, in line with GTD follow-up protocols.

## Discussion

Anaesthetic implications of complete hydatidiform mole

Complete hydatidiform mole poses several anaesthetic challenges. Uterine overdistension and abnormal trophoblastic vascularity predispose patients to sudden and potentially severe haemorrhage during evacuation [1,8]. Cardiovascular compromise may arise from acute blood loss, high-output circulatory states, or pulmonary complications related to trophoblastic embolisation or fluid shifts [9,10]. Endocrine dysfunction, particularly thyrotoxicosis, further complicates perioperative management by amplifying sensitivity to catecholamines and reducing cardiovascular reserve [5,7].

Although thyroid storm is uncommon in modern practice, it is well described in molar pregnancy and has been linked to high β-hCG concentrations, with surgical stress and anaesthetic-related sympathetic stimulation representing modifiable perioperative triggers that underscore the anaesthesiologist’s role in prevention and control [7]. Importantly, biochemical thyrotoxicosis without overt clinical features may still confer increased perioperative risk, particularly with respect to arrhythmogenesis and haemodynamic instability [5,7].

Thyroid risk assessment and cardiovascular considerations

In this patient, thyroid function testing demonstrated suppressed TSH and elevated free T4, consistent with biochemical thyrotoxicosis. However, the absence of clinical features such as tachycardia, hyperthermia, tremor, heart failure, or altered mental status, together with a low Burch-Wartofsky score, indicated a low likelihood of thyroid storm and supported classification as compensated thyrotoxicosis [7].

The presence of frequent premature ventricular complexes raised additional concerns. Ventricular ectopy in the setting of a normal structural heart and preserved ventricular function is generally considered benign; however, thyrotoxic physiology can reduce the threshold for arrhythmias, and careful control of adrenergic stimulation remains a key perioperative priority [10,12], particularly when electrolyte balance, oxygenation, and heart rate are controlled [7,11]. Sympathetic surges are recognised triggers for ventricular arrhythmias in hyperthyroid states, reinforcing the importance of minimising adrenergic stimulation during anaesthesia [5,7].

General anaesthesia: benefits and risks

GA remains the most commonly reported technique for molar evacuation, particularly in patients with large uterine size, haemodynamic instability, respiratory compromise, or suspected metastatic disease [8-10]. The ability to secure the airway and rapidly escalate surgical management confers clear advantages in high-risk scenarios.

However, GA is associated with several physiological perturbations that may be undesirable in thyrotoxic patients. Induction and airway manipulation provoke catecholamine release, which can precipitate tachycardia, hypertension, and arrhythmias [7,11]. Volatile anaesthetic agents may cause myocardial depression and vasodilation, potentially destabilising patients with reduced cardiovascular reserve [11]. Furthermore, indirect-acting vasopressors such as ephedrine increase heart rate and myocardial oxygen demand, effects that are magnified in hyperthyroid physiology [5,7].

Rationale for spinal anaesthesia

Spinal anaesthesia was selected after a structured assessment across key decision domains. Endocrine: despite biochemical thyroid dysfunction, she was clinically euthyroid, and her Burch-Wartofsky score was 0, placing her in the "unlikely thyroid storm" category (<25). Cardiovascular: she was haemodynamically stable at baseline, with preserved ventricular function, no clinical evidence of heart failure, and ectopy not associated with symptoms or instability. Haemorrhagic: significant bleeding was anticipated based on uterine size and pre-operative assessment, with cross-matched blood immediately available and a plan for rapid escalation. Airway: there was no anticipated difficult airway, but a clear pathway for prompt conversion to GA was in place should haemorrhage or cardiopulmonary compromise occur. Uterine size at 16 weeks’ gestation suggested a lower risk of catastrophic haemorrhage compared with giant moles described in the literature [8,9].

The principal concern with spinal anaesthesia in this setting is the potential for sudden sympathectomy-induced hypotension and loss of compensatory vascular tone, which may be catastrophic if unrecognised haemorrhage occurs [12]. In this patient, careful pre-operative assessment suggested a low to moderate risk of massive bleeding; haemoglobin was acceptable, uterine size was not extreme, and there were no signs of coagulopathy. Invasive arterial monitoring was used to provide continuous haemodynamic feedback, and blood products were immediately available.

Neuraxial anaesthesia avoids airway manipulation and blunts the neuroendocrine stress response to surgery, thereby reducing catecholamine release [10,12]. In patients with thyrotoxic physiology, this attenuation of adrenergic stimulation is particularly advantageous [5,7]. Case reports and small series have demonstrated the feasibility of neuraxial techniques in GTD when appropriate precautions and immediate access to GA are available [10,12].

Management of spinal-induced hypotension and vasopressor choice

Hypotension due to sympathetic blockade remains the principal limitation of spinal anaesthesia [12]. In thyrotoxic patients, hypotension may provoke reflex tachycardia, increasing myocardial oxygen consumption and arrhythmogenic risk [7]. Consequently, proactive haemodynamic control is essential.

Phenylephrine was selected as the primary vasopressor because it is a pure α-adrenergic agonist that increases systemic vascular resistance without direct β-adrenergic stimulation [11-13]. This pharmacodynamic profile allows restoration of arterial pressure while avoiding tachycardia, an important consideration in patients with ventricular ectopy or heightened β-adrenergic sensitivity [7,11]. By maintaining blood pressure within 10% of baseline, the infusion likely prevented reflex sympathetic activation and thereby limited ectopic activity. Intra-operatively, bigeminy did not worsen or evolve, which aligned with the intended physiological strategy.

In contrast, ephedrine exerts indirect β-adrenergic effects and has been associated with tachyarrhythmias in hyperthyroid states [5,7]. Obstetric and regional anaesthesia literature increasingly supports phenylephrine as the vasopressor of choice when heart rate control is desirable [13].

Risk-based selection of spinal versus general anaesthesia

Based on available evidence and this case, spinal anaesthesia may be appropriate in molar pregnancy when the following criteria are met: (a) compensated biochemical thyrotoxicosis with low thyroid storm risk [7]; (b) haemodynamic stability and preserved ventricular function [7]; (c) absence of significant respiratory compromise or suspected metastases [8-10]; (d) anticipated low-to-moderate haemorrhage risk based on uterine size and imaging [8]; and (e) immediate availability of blood products, vasopressors, invasive monitoring, and conversion to GA [10,12].

Our findings help to substantiate the claim that spinal anaesthesia can be haemodynamically and metabolically safe in this context when meticulous monitoring and vasopressor support are employed.

Published experience with neuraxial anaesthesia in molar pregnancy remains limited but broadly concordant with the present report. Solak and Aktürk described successful spinal anaesthesia in a patient with hyperthyroidism secondary to hydatidiform mole, emphasising the benefit of avoiding airway stimulation [14]. Kabade and colleagues reported a case of spinal anaesthesia in molar pregnancy with thyrotoxicosis, again with stable haemodynamics [15]. Ryalino et al. and Rajagopal et al. have similarly suggested that neuraxial anaesthesia may be preferable in hyperthyroid GTD patients without major haemorrhage or cardiopulmonary compromise [10,12]. Rajagopal et al. have argued that anaesthetic technique in GTD should be individualised, based on endocrine status, haemodynamic stability, and institutional readiness rather than solely on diagnosis [10].

However, GA remains preferable in patients with overt thyrotoxicosis, haemodynamic instability, massive uterine enlargement, respiratory compromise, or a high likelihood of hysterectomy [8-10].

## Conclusions

This case demonstrates that spinal anaesthesia can be a safe and rational choice for uterine evacuation in a patient with complete molar pregnancy, biochemical thyrotoxicosis, and ventricular ectopy, when clinical thyroid status is stable, cardiac structure and function are preserved, and haemodynamic monitoring and vasopressor strategies are optimised. GA remains entirely appropriate and often preferable in many molar pregnancy cases, particularly when airway control or rapid escalation for haemorrhage is anticipated. Rather than treating GA as obligatory, clinicians should use a physiology-based, individualised approach that balances endocrine, cardiovascular, haemorrhagic, and airway risks. When neuraxial anaesthesia is chosen, it should be supported by invasive monitoring, immediate blood product availability, and a defined plan for prompt conversion to GA if conditions deteriorate. Further multicentre observational work is needed to define evidence-based selection criteria and strengthen the limited current literature.
